# Abnormal intra- and inter-network functional connectivity of brain networks in early-onset Parkinson’s disease and late-onset Parkinson’s disease

**DOI:** 10.3389/fnagi.2023.1132723

**Published:** 2023-03-24

**Authors:** Fan Zhou, ChangLian Tan, Chendie Song, Min Wang, Jiaying Yuan, Yujing Liu, Sainan Cai, QinRu Liu, Qin Shen, Yuqing Tang, Xu Li, Haiyan Liao

**Affiliations:** Department of Radiology, The Second Xiangya Hospital, Central South University, Changsha, China

**Keywords:** late-onset Parkinson’s disease, early-onset Parkinson’s disease, independent component analysis, brain network, UPDRS-III, MMSE

## Abstract

**Objective:**

The purpose of this study is to look into the altered functional connectivity of brain networks in Early-Onset Parkinson’s Disease (EOPD) and Late-Onset Parkinson’s Disease (LOPD), as well as their relationship to clinical symptoms.

**Methods:**

A total of 50 patients with Parkinson’ disease (28 EOPD and 22 LOPD) and 49 healthy controls (25 Young Controls and 24 Old Controls) were admitted to our study. Employing independent component analysis, we constructed the brain networks of EOPD and Young Controls, LOPD and Old Controls, respectively, and obtained the functional connectivity alterations in brain networks.

**Results:**

Cerebellar network (CN), Sensorimotor Network (SMN), Executive Control Network (ECN), and Default Mode Network (DMN) were selected as networks of interest. Compared with their corresponding health controls, EOPD showed increased functional connectivity within the SMN and ECN and no abnormalities of inter-network functional connectivity were found, LOPD demonstrated increased functional connectivity within the ECN while decreased functional connectivity within the CN. Furthermore, in LOPD, functional connectivity between the SMN and DMN was increased. The functional connectivity of the post-central gyrus within the SMN in EOPD was inversely correlated with the Unified Parkinson’s Disease Rating Scale Part III scores. Age, age of onset, and MMSE scores are significantly different between EOPD and LOPD (*p* < 0.05).

**Conclusion:**

There is abnormal functional connectivity of networks in EOPD and LOPD, which could be the manifestation of the associated pathological damage or compensation.

## Introduction

Parkinson’s disease (PD) is one of the most common neurodegenerative diseases in clinical practice (Samii et al., 2004). According to the age of onset, it can be divided into the following two subtypes: Early-Onset Parkinson’s Disease (EOPD) and Late-Onset Parkinson’s Disease (LOPD). Although the cut-off age for EOPD and LOPD varies in different studies, the majority of studies use 50 years as the cut-off age. The international Parkinson and Movement Disorder Society Task Force on Early-Onset Parkinson’s Disease also recommend using 50 years as the cut-off age (Mehanna et al., 2022). EOPD is usually defined as PD patients first experiencing motor symptoms at and before the age of 50 (Butterfield et al., 1993; Fereshtehnejad et al., 2014; Hua et al., 2022). Correspondingly LOPD usually refers to PD patients who first develop motor symptoms after the age of 50. EOPD, unlike typical LOPD, has its own characteristics in terms of clinical presentation (Ferguson et al., 2016; Angelopoulou et al., 2022). Patients with EOPD usually have a longer duration and slower disease progression, but die at an earlier age, have more complications related to dopamine medication, have a relatively greater impact of the disease on their life, work and family, and are more prone to anxiety and depression (Wickremaratchi et al., 2009; Fereshtehnejad et al., 2014; Ou et al., 2021; Zhou et al., 2022). Besides, EOPD is associated more frequently with known mutations in genes linked to PD (Li et al., 2020; Zhao et al., 2020) whereas LOPD is believed to be more multifactorial. The above-mentioned manifestations suggest a different pathogenesis between EOPD and LOPD. However, the neurophysiological pathogenesis of EOPD and LOPD is still unclear.

Previous PET metabolic imaging (Sasannezhad et al., 2017), structural magnetic resonance (Xuan et al., 2019), and quantitative magnetic analysis (Xuan et al., 2017) studies have confirmed the differences in structural and metabolic alterations between EOPD and LOPD. Another useful technique for studying PD is resting-state magnetic resonance imaging (Rs-fMRI) (Filippi et al., 2018; Tessitore et al., 2019), but only a few resting-state studies have included EOPD. The following methods have been used in previous resting-state MRI studies on EOPD and LOPD: regional homogeneity (ReHo), amplitude of low frequency fluctuation (ALFF), seed -based functional connectivity, and degree centrality. A study based on ReHo and ALFF found that EOPD and LOPD have different ReHo and ALFF alterations in nodes of motor, emotional, and visual loops (Yue et al., 2020). A seed-based functional connectivity study found differences in striatal connectivity patterns between EOPD and LOPD (Hou et al., 2016). A study based on Degree Centrality (DC) found opposite trends in DC value changes at key nodes of default mode network (DMN) in EOPD and LOPD (Wang et al., 2020). The above studies suggest that there are differences in brain activity levels between EOPD and LOPD in the resting state, but ReHo and ALFF are limited to investigate changes of brain activity in local brain regions, the seed-based functional connectivity require a predetermined seed and DC only focus on the importance of particular brain regions, resulting in differences between results and poor reproducibility.

Functional brain activity is achieved by several different brain networks (Herbet and Duffau, 2020). As no prior assumptions are required, independent component analysis (ICA) can decompose Rs-MRI data into multiple independent components which are called networks as well (Calhoun et al., 2001) and enables the exploration of functional connectivity changes at the brain network level. Compared to above studies, ICA is able to explore functional connectivity changes at a larger brain scale and can avoid the selection bias associated with seed selection. In this study, we attempted to explore the changed functional connectivity of brain networks in EOPD and LOPD through ICA to find possible neuroimaging markers of them.

## Materials and methods

### Participants

A total of 85 PD patients were recruited through the department of neurology, The Second Xiangya Hospital from August 2018 to August 2021, meanwhile 66 healthy controls (HCs) were recruited in the community and outpatient clinics. The research was approved by the hospital’s ethics committee and an informed consent form was signed by each participant. Inclusion criteria for PD include: (1) Diagnosis of Parkinson’s disease based on the 2015 Movement Disorders Association diagnostic criteria for Parkinson’s disease (Postuma et al., 2015). (2) Age at first motor symptoms > 20 years. (3) Drug-Naive PD. (4) Right-handedness. (5) Modified H-Y stage ≤ 3.0. (6) No Intelligence impairment as evaluated by MMSE (Li et al., 2016): MMSE score > 17 for illiterate participants, >20 for grade-school literate participants, and >23 for junior high school and higher education literate participants were defined as normal intelligence in our participants. Exclusion criteria for PD include: (1) Parkinsonism caused by other conditions other than Parkinson’s disease, such as traumatic brain injury and drugs. (2) Other neurological and psychiatric disorders, such as schizophrenia, epilepsy. (3) Severe injury of the cerebrum, such as extensive cerebral infarction, hemorrhagic stroke, and brain tumor. (4) Long-term alcohol and drug abuse. (5) The presence of absolute contraindications to MRI. (6) Failure to complete the clinical scale assessment. The inclusion criteria for healthy controls include: (1) Voluntary participation in this study. (2) Right-handedness. (3) No intelligence impairment. Exclusion criteria for HCs include: the same as exclusion criteria of (2) to (5) for the PD group. After screening the recruited candidates, 50 HCs and 52 PD patients were initially enrolled in this research.

### Clinical scale assessment

All clinical scales were assessed by the same neurologist who specializes in Parkinson’s disease on the same day as the MRI examination was performed. The clinical scales included the Mini-Mental State Examination (MMSE) to evaluate cognitive status, the Unified Parkinson’s Disease Rating Scale (UPDRS) to evaluate clinical symptoms in PD, and the Modified Hoehn-Yahr (H-Y) staging scale to assess the severity of PD.

### Magnetic resonance data acquisition

A Siemens 3.0 T MRI machine (MAGNETOM Skyra, Germany) was used to collect all MRI images. The MRI images consist of rs-fMRI images and three-dimensional T1-weighted structural MRI images. During the scans, Participants wore earplugs to avoid noise disturbance. Foams were put on each side of the head to reduce head movement. They remained awake during the scans. Parameters are as follows, Rs-fMRI: Number of layers = 39, Thickness of layer = 3.5 mm, TR = 2,500 ms, TE = 25 ms, FA = 90°, FOV = 240 × 240 mm, Acquisition matrix = 64 × 64, Voxel size = 3.8 × 3.8 × 3.5 mm, Whole brain volume = 200. Structural parameters: Number of layers (Sagittal) = 176, Thickness of layer = 1.0 mm, TR = 1900.0 ms, TE = 2.01 ms, FA = 9°, FOV = 256 × 256 mm.

### Data preprocessing

RESTplus was used to preprocess the raw MRI data on MATLAB 2014a. The steps included: (1) Format Conversion and removal of the first 10 volumes of Rs-fMRI images: The format of images is converted from DICOM to NIFTI and 190 of 200 volumes were preserved. (2) Slice timing. (3) Head motion correction: those with horizontal head movement > 0.5 mm or rotation angle > 0.5°were excluded. (4) Spatial normalization: Rs-fMRI images were normalized to Montreal Neurological Institute (MNI) template by dartel using the T1 image new segment. The resampled voxel size is 3 × 3 × 3 mm. (5) Spatial Smoothing: The used Gaussian kernels is 6 mm FWHM.

### Brain networks construction and selection of networks of interest

**FIGURE 1:**
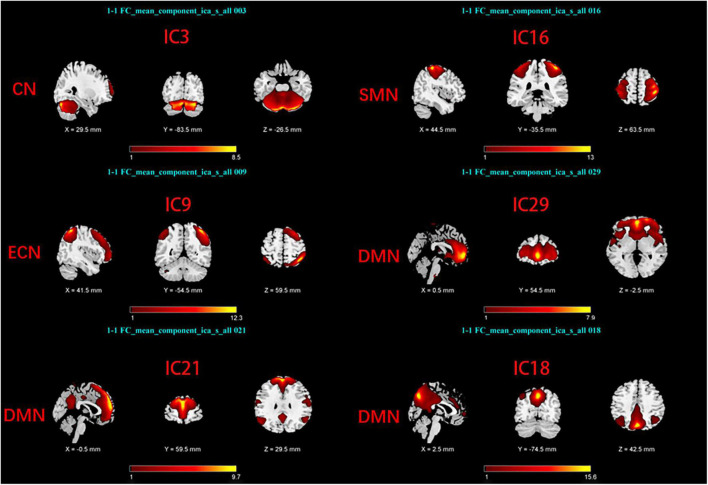
Spatial maps of brain networks of EOPD and YCs. SMN, sensorimotor network; DMN, default mode network; ECN, executive control network; CN, cerebellar network.

**FIGURE 2:**
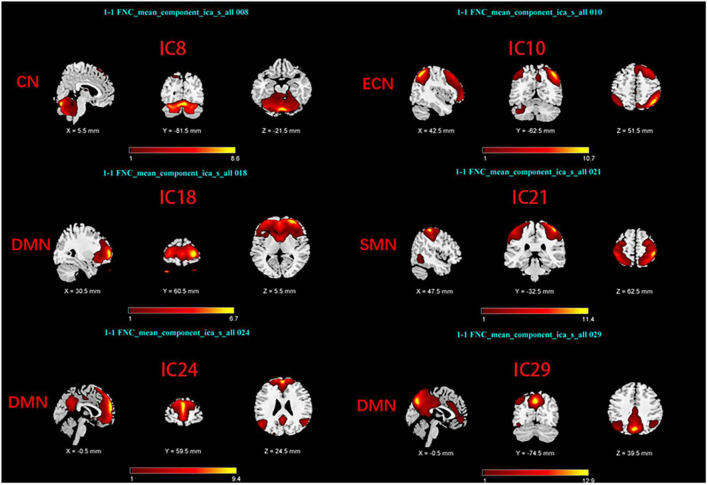
Spatial maps of brain networks of LOPD and OCs. SMN, sensorimotor network; DMN, default mode network; ECN, executive control network; CN, cerebellar network.

Group Independent Component Analysis Toolbox (GIFT4.0b)^1^ was used to process the Rs-fMRI data. We chose spatial ICA for our data analysis. Firstly, Component numbers estimation for EOPD and young controls (YCs), and for LOPD and old controls (OCs) were performed using the MDL criteria (Rissanen, 1983). Secondly, Rs-fMRI data were decomposed into several components, the number of which was consistent with the mean numbers automatically estimated by GIFT (Liao et al., 2020; Liu et al., 2022): 29 components for EOPD and YCs, and 30 components for LOPD and OCs. 20 times ICASSO were run to ensure the stability of the component decomposition according to previous literature (Kim et al., 2017). The algorithm for decomposition was Infomax (Bell and Sejnowski, 1995). The main process of component decomposition includes two-step data reduction and back-reconstruction using GICA (Du and Fan, 2013). All results were converted to Z-Scores. Finally, the spatial z-map and time course for each component were obtained. We selected the brain network of interest in two steps: (1) Template-matching: Spatial correlation analysis was performed between spatial z-maps of components and templates (Shirer et al., 2012). (2) Visual inspection: Components were selected as meaningful networks in line with previous literature’s standards (Cordes et al., 2000; Allen et al., 2014). After template matching and visual inspection, six components were selected as brain networks of interest in each of the two groups, including the sensorimotor network (SMN), the cerebellar network (CN), the default mode network, and the executive control network (ECN). The components of the two groups correspond to the brain networks as follows: In EOPD and YCs, SMN (IC16), DMN (IC18, 21, 29), CN (IC3), and ECN (IC9) (Figure 1). LOPD and OCs: SMN (IC21), DMN (IC18, 24, 29), CN (IC8), and ECN (IC10) (Figure 2).

### Statistical analysis

Statistical analysis of demographic data as well as clinical data was performed on SPSS v22.0 software.

To obtain altered functional connectivity within networks, The spatial maps of components in EOPD and YCs, LOPD and OCs were subjected to a one-sample *t*-test separately by using SPM12 (*P* < 0.001 at voxel level and cluster size > 13 voxels correspond to a corrected *p* < 0.05 determined by AlphaSim correction). Then two-sample *t*-test was conducted, respectively, on the spatial maps between EOPD and YCs, between LOPD and OCs with age and sex as covariates (*P* < 0.05, Alphasim corrected).

Detrending, Despiking, and Temporal filtering were conducted on the time course of components before inter-network functional connectivity analysis. Among the components, Pearson’s correlation coefficients of the time course, which represent inter-network functional connectivity, were calculated. All of them were converted into z-scores. We then performed a two-sample *t*-test on z-scores to obtain inter-network functional connectivity differences between EOPD and YCs, and between LOPD and OCs (FDR corrected, *p* < 0.05). Age and sex were used as covariates. All steps are implemented in the MANCOVAN of the GIFT.

In EOPD and LOPD, analysis of correlations was performed between brain regions which showed altered intra-networks functional connectivity and the scores of MMSE, the total score of UPDRS, and the scores of UPDRS-III, and multiple comparison correction was performed. The UPDRS-III is the third part of UPDRS that deals with motor symptoms.

## Results

### Demographic data and clinical data characteristics

After removing 3 subjects due to head movement (1 healthy control, 2 PD patients), ultimately 50 patients with PD (28 EOPD and 22 LOPD) and 49 healthy controls (25 YCs and 24 OCs) were included in this study. Demographic and clinical data showed no significant differences between EOPD and YCs, and between LOPD and OCs. Significant differences in age, age of onset, and MMSE scores were found between EOPD and LOPD (*p* < 0.05). The demographic data and clinical data characteristics are listed in Table 1.

**TABLE 1:** Demographic and clinical information of patients with Parkinson’s disease and healthy controls.

	EOPD	YCs	LOPD	OCs	*P*-value		
					**EOPD VS. YCs**	**LOPD VS. OCs**	**EOPD VS. LOPD**
Gender (Female)	28 (13)	25 (18)	22 (12)	24 (12)	0.06	0.758	0.569
Age^a^	47.25 ± 5.64	48.64 ± 2.87	58.22 ± 3.29	58.37 ± 3.80	0.258	0.889	<0.001*
Age of Onset^b^	44.53 ± 4.84	NA	55.86 ± 3.41	NA	NA	NA	<0.001*
Duration^b^, years	2.78 ± 2.83	NA	2.35 ± 1.57	NA	NA	NA	0.929
Education^a^, years	8.32 ± 3.38	9.04 ± 2.96	7.36 ± 4.39	7.22 ± 3.61	0.418	0.91	0.388
Modified H-Y stage^b^	1.68 ± 0.63	NA	1.95 ± 0.57	NA	NA	NA	0.146
Total scores of UPDRS^b^	26.25 ± 17.37	NA	34.59 ± 21.45	NA	NA	NA	0.137
Scores of UPDRS-III^b^	16.57 ± 11.55	NA	22.64 ± 15.50	NA	NA	NA	0.177
MMSE^b^	27.68 ± 2.07	28.08 ± 2.34	25.41 ± 2.71	25.87 ± 3.50	0.199	0.513	0.004*

All data are presented as Median ± SD. EOPD, early-onset Parkinson’s disease; LOPD, late-onset Parkinson’s disease; YCs, young controls; OCs, old controls; H-Y stage, Hoehn and Yahr stage; UDPRS, Unified Parkinson’s Disease Rating Scale; MMSE, Mini-Mental State Examination; UDPRS-III, the third part of Unified Parkinson’s Disease Rating Scale. The chi-square test was performed for comparing gender differences between groups, Other data were compared between groups using two-sample *t*-test or Mann–Whitney U test.

^a^Represents two-sample *t*-test.

^b^Represents Mann–Whitney U test.

*Represent a significance at *P* < 0.05.

### Functional connectivity analysis

**FIGURE 3:**
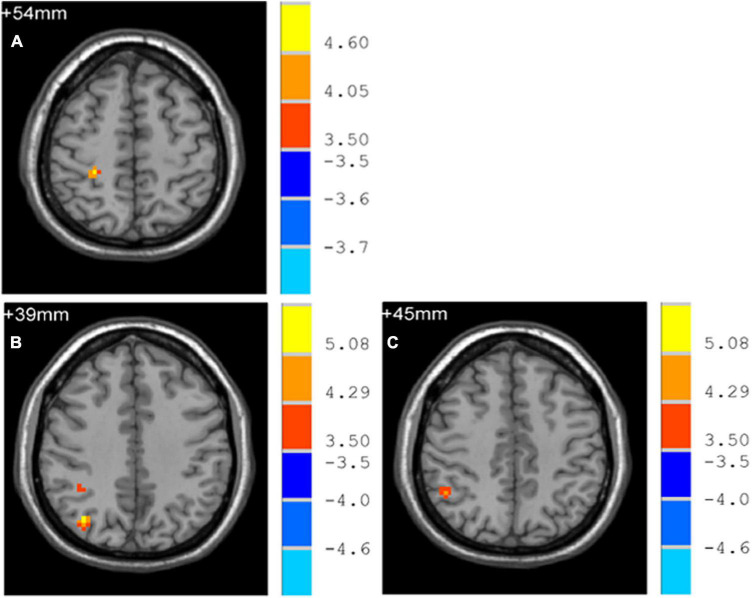
Brain regions with altered intra-networks functional connectivity in EOPD. **(A)** Right post-central gyrus. **(B)** Right angular gyrus. **(C)** Right inferior parietal gyrus. Yellow and red represent brain regions with significantly increased intra-networks functional connectivity in EOPD. Two-sample *t*-tests results are presented (*p* < 0.05, AlphaSim corrected).

**FIGURE 4:**
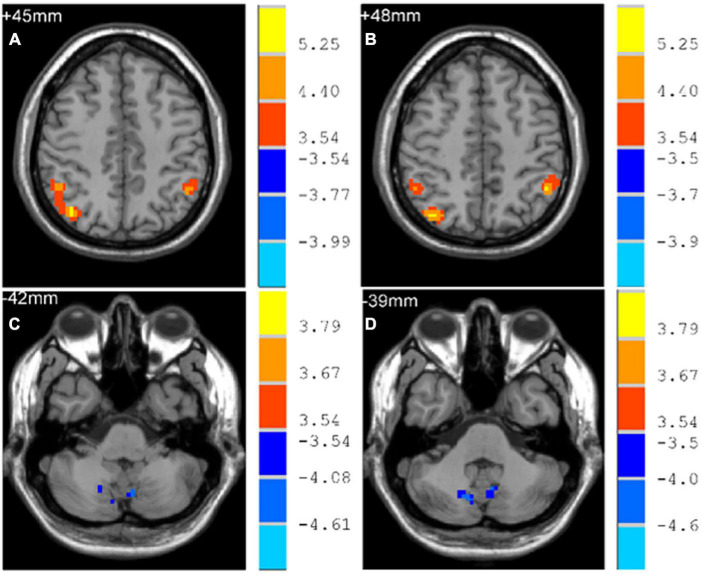
Brain regions with altered intra-networks functional connectivity in LOPD. **(A)** Right angular gyrus. **(B)** Left inferior parietal gyrus. **(C)** Lobule VIII of left cerebellar hemisphere. **(D)** Lobule VIII of right cerebellar hemisphere. Yellow and red represent brain regions with significantly increased intra-networks functional connectivity in LOPD. Blue represents brain regions with significantly decreased intra-networks functional connectivity in LOPD. Two-sample *t*-tests results are presented (*p* < 0.05, AlphaSim corrected).

**FIGURE 5:**
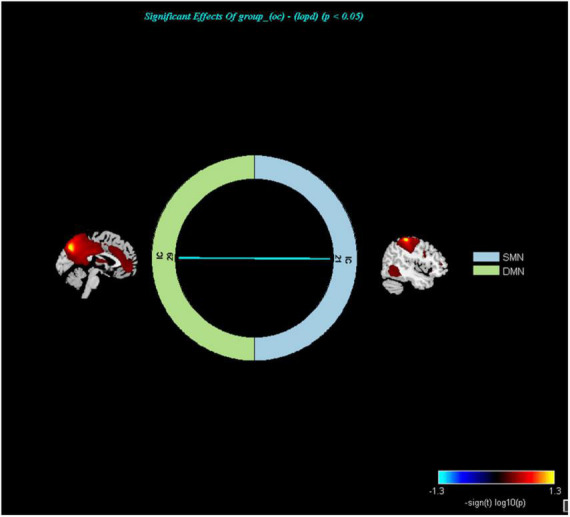
Networks with altered inter-network functional connectivity between LOPD and OCs (*P* < 0.05, FDR corrected). Color represents functional connection strength. The cooler the color, the weaker the functional connection. SMN, sensorimotor network; DMN, default mode network.

Early-Onset Parkinson’s Disease demonstrated increased functional connectivity of the right post-central gyrus within the SMN, as well as the right angular gyrus and the right inferior parietal gyrus within the ECN, when compared to YCs (Figure 3 and Table 2). No significantly reduced functional connectivity within networks were found in EOPD. LOPD demonstrated greater functional connectivity of the left inferior parietal gyrus and the right angular gyrus within the ECN and decreased functional connectivity of bilateral Lobule VIII of the cerebellar hemisphere within the CN when compared to OCs (Figure 4 and Table 2). No significant enhancement or weakening of inter-network functional connectivity was seen in EOPD compared to YCs. There was increased functional connectivity between the DMN and the SMN in LOPD compared to OCs (Figure 5), but no decreased functional connectivity between networks was found in LOPD. Both EOPD and LOPD didn’t show altered functional connectivity within the DMN, EOPD didn’t show altered functional connectivity within the CN, LOPD didn’t show altered functional connectivity within the SMN. In EOPD and LOPD, All brain regions that present altered functional connectivity within the ECN belong to the inferior parietal lobule (IPL).

**TABLE 2:** Brain regions with altered intra-network functional connectivity in EOPD and LOPD.

Brain regions (AAL)	Voxel size	Peak MNI coordination (X, Y, Z)			*T*-value
**EOPD > YCs**					
Postcentral_R	20	27	−36	54	5.151
Angular_R	27	36	−72	39	5.873
Parietal_Inf_R	19	48	−51	45	4.613
**LOPD > OCs**					
Angular_R	106	39	−69	45	6.110
Parietal_Inf_L	32	−48	−51	48	5.460
**LOPD < OCs**					
Cerebellum_8_L	14	−6	−69	−42	−4.345
Cerebellum_8_R	17	12	−72	−39	−4.527

### Correlation analysis

**FIGURE 6:**
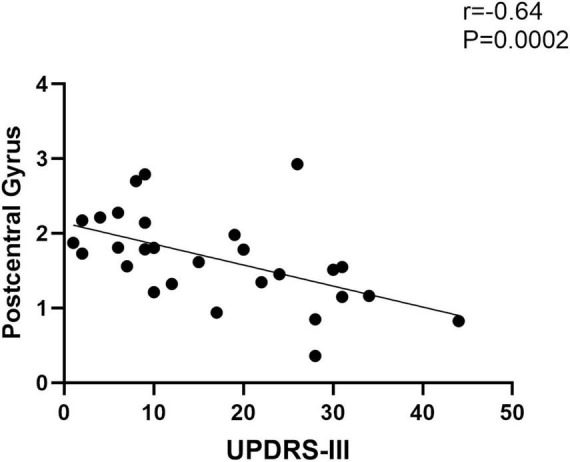
Functional connectivity of the post-central gyrus within the sensorimotor network in EOPD is negatively correlated with UPDRS-III scores (Spearman correlation; *P* < 0.05/3, Bonferroni corrected).

The functional connectivity of the right post-central gyrus within SMN in EOPD was negatively correlated with UPDRS-III scores (Figure 6) (Spearman correlation; *P* < 0.05/3 = 0.017, Bonferroni corrected). No significant correlation was found between other brain regions with altered intra-network functional connectivity and clinical scales.

## Discussion

We employed ICA to compare and analyze networks functional connectivity changes (both intra- and inter- networks) in patients with EOPD and LOPD, to explore functional connectivity alterations of brain networks in both groups and to find possible neuroimaging markers in this research. Our findings are as follows.

### Enhanced functional connectivity within executive control network may be a common compensatory functional alteration in both EOPD and LOPD

The functions of the ECN include task switching which are driven by intrinsic orientation and external stimuli (Murphy et al., 2020), goal-oriented cognition, working memory and so on. The ECN is primarily located anatomically in the frontal and parietal lobes. In our research, the functional connectivity of the inferior parietal lobe (IPL) within the ECN was increased in both EOPD and LOPD. The position of the IPL is at the rear part of the parietal lobe which involves in motor planning and motor control (Iacoboni, 2006). PD Patients frequently experience a variety of motor symptoms, one of which is freezing of gait (FOG). In the conventional view, FOG in PD is attributed to damage to basal ganglia (Yanagisawa, 2018). However, Brain regions, such as the parietal lobule, are also associated with the production of FOG (Crémers et al., 2012; Herman et al., 2013; Shine et al., 2013). Compared with PD patients without FOG, previous researches indicated that PD patients with FOG had less activity of the IPL (Tessitore et al., 2012) and reduced gray matter volume of IPL (Herman et al., 2014). A PET-CT based study found increased activation of the bilateral IPL in PD patients compared to HCs during continuous finger movements (Samuel et al., 1997), interpreting it as a compensatory mechanism for the damage of mesial frontal–striatal circuits. Altered functional connectivity of the IPL was also present in the non-motor study of Parkinson’s disease. A task-dependent fMRI study reported hyperactivation of the bilateral IPL during set-shifting in unmedicated PD patients compared to HCs (Gerrits et al., 2015), but there was no significant difference in performance between the two group, the researcher suggested that it may be a compensatory manifestation. Moreover, a meta-analysis of resting-state functional studies suggested the importance of the IPL in PD, Especially the right IPL (Tahmasian et al., 2017). Compared to HCs, intrinsic activity of the right IPL was increased in PD patients on unmedicated status, which was interpreted as an attempt to compensate for the dysfunction of the basal ganglia. Although researches included in this meta-analysis were not limited to a certain subgroup of PD patients, EOPD and LOPD were not included. Our study may further confirm the important role of the IPL in PD regardless of age of onset. In line with the above studies, we suggest that the inferior parietal lobe may be an important node in the ECN and the enhanced functional connectivity of ECN may be an important common compensatory functional alteration in EOPD and LOPD.

### Increased functional connectivity within the sensorimotor network may be a compensatory response to motor injury in EOPD

The SMN has a key role in motion processes and somatosensory handling (Levin et al., 2019; GóMEZ et al., 2021), with core areas including the precentral and post-central gyrus. The Primary somatosensory cortex is located at the post-central gyrus (Kropf et al., 2019), its roles include not only the processing of sensation and touch throughout the body, integration of sensory and motor information, but also the modulation of emotion. Anatomically, the basal ganglia receive sensory information from the sensory cortex and projects them to the motor cortex (Albin et al., 1989; Kaji et al., 2005). The production of motor symptoms in Parkinson’s disease is closely related to dysfunction of the cortico-basal ganglia-thalamic loop (Singh, 2018; Vicente et al., 2020). In our research, we discovered that the right post-central gyrus within the SMN had increased functional connectivity in EOPD compared with YCs, and found a negative correlation between its functional connectivity and UPDRS-III scores in EOPD. Our findings seem to support a hypothesis that basal ganglia dysfunction may lead to functional alterations in the sensorimotor system. Some studies also find altered functional connectivity of post-central gyrus in PD. For instance, a meta-analysis of the cortico-basal ganglia-thalamic loop showed enhanced functional connectivity in the left post-central gyrus in PD patients, and the finding wasn’t affected by medication (Ji et al., 2018). Another fMRI study reported that in PD patients responding to repetitive transcranial magnetic stimulation (rTMS), rTMS not only increased degree centrality in the post-central gyrus after rTMS but also decreased UPDRS-III scores (Chi et al., 2022). The researcher concluded that sensorimotor network was involved in the motor improvement following rTMS treatment. Meanwhile, they also found a negative correlation between baseline degree centrality values of left post-central gyrus and motor improvement, and that baseline degree centrality in the left post-central gyrus was able to differentiate the responders of rTMS from non-responders in PD patients. Combined with the above studies, we suggest that increased functional connectivity within the SMN may be a compensatory response to motor injury in EOPD, and the post-central gyrus may be an important node in the sensorimotor network.

### Reduced functional connectivity within the cerebellar network may be a neuroimaging marker of cerebellar functional impairment in LOPD

In our research, Compared with OCs, LOPD displayed reduced functional connectivity of bilateral Lobule VIII of the cerebellar hemisphere within the CN. Functional MRI studies have confirmed cerebellar Lobule VIII activation no matter in motor or in non-motor tasks (Stoodley and Schmahmann, 2009; Balsters et al., 2014; E et al., 2014). The cerebellum is involved in numerous functions (Stoodley and Schmahmann, 2010; Buckner, 2013; Steele et al., 2017), such as motor control, emotional processing, working memory. By means of PET-CT, fMRI and structural MRI, researchers have found that there are metabolic, functional as well as structural alterations in the cerebellum of PD patients and such alterations are associated with the production of motor and non-motor symptoms of PD (Wu and Hallett, 2013; Haghshomar et al., 2022; Zhong et al., 2022), especially tremor and cognitive impairment. A PET-based study revealed a tremor-related metabolic network in PD which showed increased activity of the cerebellum, primary motor cortex and caudate/putamen and correlated significantly and positively with clinical ratings of tremor. The researcher suggested that tremor is mediated by a distinct metabolic network involving cerebello-thalamo-cortical loop in PD (Mure et al., 2011). A PD-related cognitive metabolic network which showed increased metabolism in cerebellum and decreased metabolism in frontal and parietal areas was found in non-demented PD patients. It is correlated with cognitive performance and the researcher suggest that the increased metabolism of cerebellum may be a compensation alteration to the loss of dopaminergic input to the striatum (Huang et al., 2007). Both of them suggest an association of the cerebellum with Parkinson’s disease. Combining the perspectives of the above studies, we suggest that reduced functional connectivity within the CN may be a manifestation of cerebellar damage in neural activity level and may be a useful neuroimaging marker to indicate the cerebellar functional damage in LOPD.

In addition, increased inter-networks functional connectivity was found between the DMN and SMN in LOPD, suggesting that enhanced collaboration between the two networks may be one of the compensatory neurophysiological mechanisms in LOPD. Previous studies also found altered inter-network functional connectivity in PD patients (Peraza et al., 2017; Caspers et al., 2021).

Age and MMSE scores were significantly different between EOPD and LOPD in our study. Since there is a difference between EOPD and LOPD in age of onset and no significant difference in disease duration, it is understandable that there is a difference in age between the two groups. We did not find significant correlations between MMSE scores and brain areas with altered functional connectivity within the networks of EOPD and LOPD, and due to the small number of clinical scales, whether the difference in MMSE scores between the two groups is due to other factors is unclear.

There is still some room for improvement in our study. Firstly, the sample size and the number of clinical scales of this study are small, which to some extent limits the application value of this study. We will address this issue by recruiting more participants and adding more scales subsequently. Secondly, PD patients included in our research have a relatively short disease course and all of them are in the early to mid-stage of this disease (H-Y stage ≤ 3). Functional connectivity changes in some brain networks may not be apparent at this time. In a previous study, we investigated different patterns of altered brain activity in PD at different stages of the disease, and found that the higher the grade, the more extensive the involvement of the brain (Li et al., 2021). Thirdly, our study is a cross-sectional study. Whether brain network alterations will change with time or treatment is still unknown, We will supplement this with follow-up visits.

In summary, We find altered functional connectivity of brain networks in both EOPD and LOPD, and there are similarities and distinctions between EOPD and LOPD in regard to functional connectivity changes within and between brain networks, which could be the manifestation of the associated pathological damage or compensation, and further research is needed. The above findings suggest that altered functional connectivity of brain networks may be a potential neuroimaging marker for EOPD and LOPD and provide an important reference point for subsequent studies.

## Data availability statement

The raw data supporting the conclusions of this article will be made available by the authors, without undue reservation.

## Ethics statement

The studies involving human participants were reviewed and approved by the Ethics Committee of the Second Xiangya Hospital of Central South University. The patients/participants provided their written informed consent to participate in this study.

## Author contributions

FZ, CS, MW, JY, YL, SC, QL, QS, YT, and XL: data collection. FZ and HL: data analysis. FZ, HL, and CT: manuscript writing. CT and HL: project development and manuscript revision. All authors contributed to the article and approved the submitted version.

## Footnotes

1
https://trendscenter.org/software/gift/
